# The topoisomerase II/condensin II axis silences transcription during germline specification in *Caenorhabditis elegans*

**DOI:** 10.1093/g3journal/jkae236

**Published:** 2024-10-03

**Authors:** Mezmur D Belew, Emilie Chien, Matthew Wong, W Matthew Michael

**Affiliations:** Department of Biological Sciences, Molecular and Computational Biology Section, University of Southern California, Los Angeles, CA 90089, USA; Department of Biological Sciences, Molecular and Computational Biology Section, University of Southern California, Los Angeles, CA 90089, USA; Department of Biological Sciences, Molecular and Computational Biology Section, University of Southern California, Los Angeles, CA 90089, USA; Department of Biological Sciences, Molecular and Computational Biology Section, University of Southern California, Los Angeles, CA 90089, USA

**Keywords:** *Caenorhabditis elegans*, germline, PIE-1, topoisomerase II, condensin, genome silencing, global transcriptional repression

## Abstract

In *Caenorhabditis elegans*, the germline is specified via a preformation mechanism that relies on the PIE-1 protein's ability to globally silence mRNA transcription in germline precursor cells, also known as the P lineage. Recent work from our group has identified additional genome silencing events in *C. elegans* during oogenesis and in starved L1 larvae, and these require the condensin II complex, topoisomerase II, and components of the H3K9me/heterochromatin pathway. Interestingly, silencing in oocytes also requires PIE-1, but this is not the case in starved L1s. Here, we ask if additional genome silencing components besides PIE-1 are required to repress gene expression in the P lineage of early embryos, and we find that condensin II and topoisomerase II are required and the H3K9me/heterochromatin pathway is not. We show that depletion of topoisomerase II/condensin II activates the normally suppressed RNA polymerase II to inappropriately transcribe somatic genes in the P lineage. We also present evidence that while both PIE-1 and topoisomerase II/condensin II are required for genome silencing in the P lineage, PIE-1 can silence transcription independently of topoisomerase II/condensin II when misexpressed in somatic cells. Thus, in oocytes, all three genome silencing systems (topoisomerase II/condensin II, H3K9me, and PIE-1) are operational, while in both early embryos and starved L1s two of the three are active. Our data show that multiple, redundantly acting genome silencing mechanisms act in a mix-and-match manner to repress transcription at different developmental stages in the *C. elegans* germline.

## Introduction

Just like individual genes, the transcriptional output of entire genomes can be regulated in a signal-mediated manner. In particular, a global repression of transcription is observed when cells enter quiescence (Swygert and Tsukiyama 2019; Miles *et al.* 2021), are under environmental stress (Kantidze *et al.* 2015), or when they experience DNA damage (Ajit *et al.* 2024; Talukdar *et al.* 2024). In many organisms, global transcriptional repression is also observed in the germline during its specification, where repression serves to maintain the germline fate (Wang and Seydoux 2013; Strome and Updike 2015). Yet another occurrence of global transcriptional repression, also known as genome silencing, occurs during oogenesis where a highly conserved feature of meiotic maturation is the silencing of the oocyte genome (Landsberger and Wolffe 1997; Bouniol-Baly *et al.* 1999; Schultz *et al.* 2018). While the phenomenon of genome silencing has been appreciated for decades, the molecular mechanisms in play are just now being resolved.

**Fig. 1.**
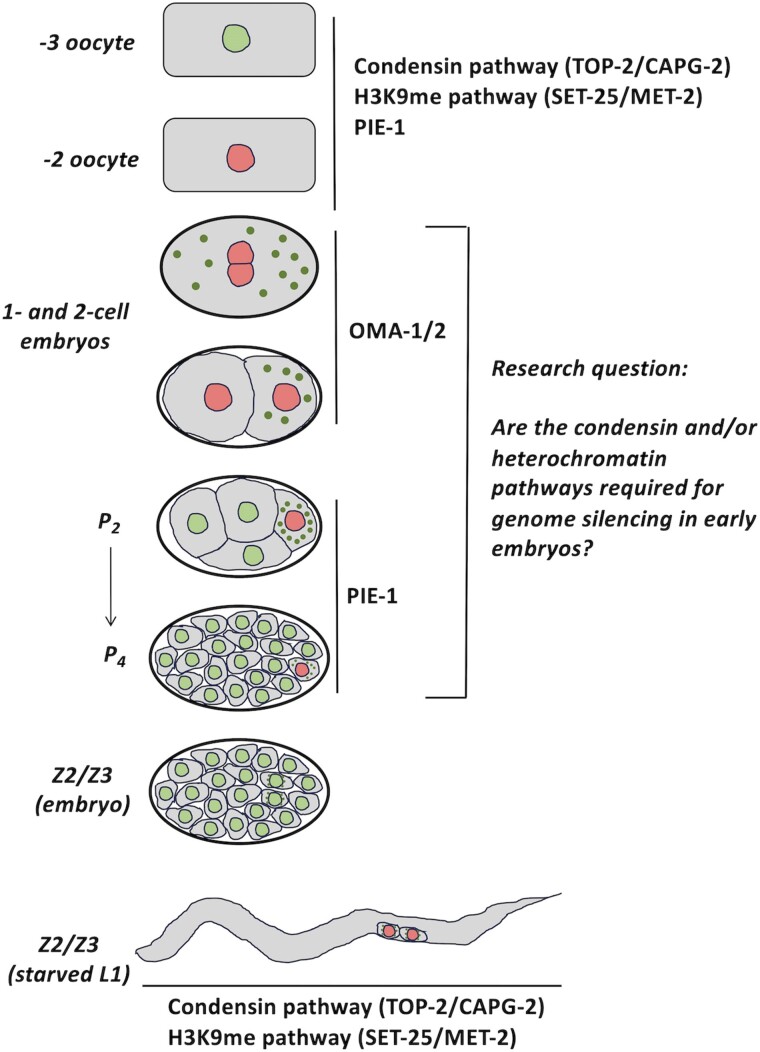
Schematic representation of known genome silencing components during *C. elegans* germline development. See text for details. Nuclei colored in red indicate transcriptionally silenced cells.

An outstanding model organism with which to study genome silencing pathways is the roundworm *Caenorhabditis elegans*. This is particularly true in the germline, where multiple genome silencing events have been documented (summarized in Fig. 1). Starting with the gametes, we and others have shown that for both oocytes and spermatocytes transcription is globally silenced as these cells exit meiotic prophase (Walker *et al.* 2007; Belew *et al.* 2023; Chien and Michael 2023). Silencing during gametogenesis requires three distinct pathways. One is comprised of topoisomerase II (TOP-2 in worms) and condensin II, which work together to compact chromatin and to silence gene expression (Belew *et al.* 2023; Chien and Michael 2023). The second is comprised of components of the H3K9me/heterochromatin pathway, for example, the methyltransferases SET-25 and MET-2, and is characterized by a large-scale buildup of H3K9me marks on chromatin as the genome is silenced (Belew *et al.* 2023). Lastly, the PIE-1 protein is also required for genome silencing during oogenesis (Belew *et al.* 2023). Upon fertilization, transcription continues to be repressed in the newly formed zygote, via the activities of the OMA-1/2 proteins, and this continues through first mitosis into the 2-cell embryo (Guven-Ozkan *et al.* 2008). After the second mitosis, which forms the 4-cell embryo, OMA-1/2 are abruptly degraded and this allows transcriptional activation in the somatic cells ABa, ABp, and EMS. The germline precursor, P_2_, remains transcriptionally repressed due to the activity of PIE-1 (Mello *et al.* 1996; Seydoux *et al.* 1996). PIE-1 continues to repress transcription during germline specification in the so-called P lineage (P_2_, P_3_, and P_4_) until P_4_ divides to form the primordial germ cells (PGCs) Z2 and Z3. Upon division of P_4_ the PIE-1 protein is instantly degraded (Mello *et al.* 1996; Schaner *et al.* 2003), and transcription is activated in the germline for the first time. Recent work from our group has shown that transcription continues in Z2/Z3 as the embryo hatches to form an L1 larva, but only if nutrients are present (Belew *et al.* 2021). If embryos hatch into a nutrient-free environment, then the energy sensor AMPK is activated and this promotes chromatin compaction and genome silencing in a manner dependent on the TOP-2/condensin II and H3K9me pathways (Belew *et al.* 2021). In this condition, PIE-1 is dispensable for genome silencing as there is no detectable PIE-1 protein present in starved Z2/Z3 (Supplementary Fig 3B). Thus, for the germline, transcription is globally repressed during gametogenesis, as well as in 1- and 2-cell embryos. Transcription remains repressed in the germline progenitor P cells prior to activation in Z2/Z3, and can then be silenced again during L1 starvation and reactivated after feeding.

The findings discussed above highlight the mechanistic plasticity in how germline transcription is controlled globally. Oocytes use TOP-2/condensin II, the H3K9me pathway, and PIE-1, and loss of any one of these pathways prompts unscheduled transcription. After fertilization, OMA-1/2 becomes essential for silencing in P_0_ and P_1_. The requirement for PIE-1 returns with the birth of P_2_, and this lasts until PIE-1 is destroyed upon division of P_4_. Later, in starved L1s, TOP-2/condensin II and the H3K9me heterochromatin pathway are required once again, and this time they act in a concerted manner. Thus, multiple systems are active in oocytes and Z2/Z3; however, in the P lineage, to date, we only know of single systems—OMA-1/2 for P_0_/P_1_ and PIE-1 for P_2_–P_4_. In this work we asked if the TOP-2/condensin II and/or the H3K9me heterochromatin pathways are required in the P lineage for genome silencing, and found that the H3K9me heterochromatin pathway is dispensable, while TOP-2/condensin II is required for P_2_–P_4_, but not for P_0_–P_1_. We also found that TOP-2/condensin II requires the germline progenitor state to silence the genome, and that PIE-1 does not. These findings increase our understanding of how whole-genome silencing occurs and highlight the importance of developmental context in regulating the ability of TOP-2/condensin II to perform this task.

## Materials and methods

### *Caenorhabditis elegans* strains

N2 (wild-type), WMM1*([pie-1::gfp::pgl-1 + unc-119(+)]; [(pAA64)pie-1p::mCherry::his-58 + unc119(+)] IV)*; WMM2*(ltls37 [(pAA64) pie-1p::mCherry::his-58 +unc-119(+)] IV; unc-4(e120) top-2(it7ts) II)*; MT17463*(set-25(n5021) III)*; WM330*(pie-1(ne4301[pie-1::GFP]) III)*; AG275*(top-2(av64)[TOP-2::3XFLAG] II);* EG5175 *(pie-1(ne4301[pie-1::gfp]) III;* and *mex-5(egx1[F294N & F339N]) IV)* strains were used in this study. Worms were maintained on 60-mm plates containing nematode growth media (NGM) seeded with the *E. coli* strain OP50 or HT115. Worms were grown at 20°C and propagated through egg preparation (bleaching) every 72 hours.

### Bacterial strains

OP50 bacteria served as the primary food source. It was grown in LB media containing 100-µg/ml streptomycin by shaking at 37°C overnight. Five hundred microliters of the culture was seeded on Petri dishes containing NGM + streptomycin. HT115 bacteria grown in LB media containing 100-µg/ml carbenicillin and 12.5-µg/ml tetracycline and seeded on NGM + carbenicillin + tetracycline plates were also used as a source of food. Our RNAi strains were obtained from the Ahringer library and verified by Sanger sequencing. Bacteria containing dsRNA were streaked on LB-agar plates containing 100-µg/ml carbenicillin and 12.5-µg/ml tetracycline and incubated at 37°C overnight. Single colonies were then picked and grown in 25-ml LB cultures with 100-µg/ml carbenicillin and 12.5-µg/ml tetracycline. Five hundred microliters of this culture was seeded on 60-mm Petri dishes containing 5-mM IPTG.

### Egg preparation

Bleach solution containing 3.675 ml H_2_O, 1.2-ml NaOCl, and 0.125 ml 10N NaOH was prepared. Adult worms were washed from plates with 5 ml of M9 minimal medium (22 mM KH_2_PO_4_, 22 mM Na_2_HPO_4_, 85 mM NaCl, and 2 mM MgSO_4_). The worms were centrifuged at 1.9krpm for 1 minute and the excess medium was removed, then the bleach solution was added. Eggs were extracted by vortexing for 30 seconds and shaking for 1 minute. This was done a total of 3 times and the worms were vortexed one last time. Then the eggs were spun down at 1900rpm for 1 minute and excess bleach solution was removed and the eggs were washed 3 times with M9 minimal medium.

### RNAi treatment

RNAi containing NGM plates were prepared as described in the “Bacterial strains” section. For double RNAi treatments, the RNAi cultures were mixed at a 1:1 ratio by volume. HT115 cells transformed with an empty pL4440 vector were used as a negative control. The RNAi conditions used in this study and tests for their efficacy are described as follows.

#### *met-2* RNAi

Worms were grown on HT115 food plates for the first 24 hours and were moved to plates containing *met-2* RNAi for the remaining 48 hours.

#### top-2 RNAi

L1 worms were plated on HT115 food plates for the first 24 hours and were then moved to plates seeded with *top-2* RNAi for the remaining 48 hours. Embryonic lethality was observed at >90%.

#### capg-2 RNAi

Worms were grown on HT115 food plates for the first 24 hours and were moved to plates containing *capg-2* RNAi for the remaining 48 hours. An embryonic lethality of 80–100% was seen with this RNAi treatment.

#### pie-1 RNAi

Worms were grown on plates containing *pie-1* RNAi for the entirety of their life cycle. An embryonic lethality of 100% was observed for this RNAi.

#### mex-5/6 RNAi

Worms were grown on HT115 food plates for the first 48 hours of their life and were then switched to *mex-5/6* RNAi plates for the remaining 24 hours. An embryonic lethality of 90–100% was observed for this RNAi treatment.

#### *mex-6*/control RNAi

Worms were grown on *mex-6*/control RNAi plates for the entirety of their life cycle. An embryonic lethality of 90–100% was observed.

#### mex-6/pie-1 RNAi

Worms were grown on *mex-6/pie-1* RNAi plates for the entirety of their life cycle. An embryonic lethality of 90–100% was observed.

### Antibodies and dilutions

**Fig. 2.**
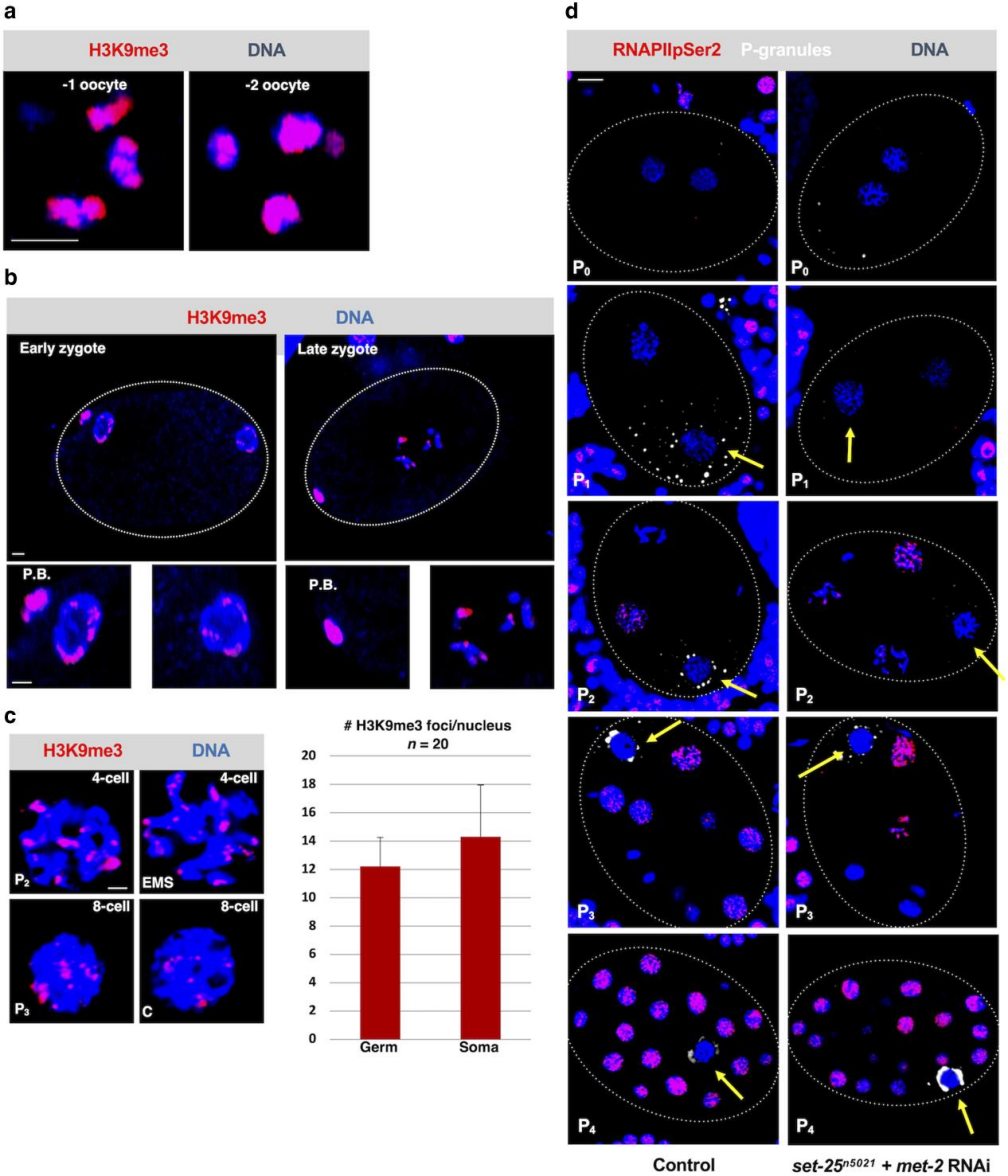
The H3K9me heterochromatin pathway is dispensable for genome silencing in P blastomeres. a) Wild-type oocytes were fixed and stained for H3K9me3 (red) and DNA (blue). Shown are magnified views of the −1 and −2 oocytes, which are the oocytes most proximal and second-most proximal to the spermatheca, respectively. The H3K9me3 signal overlaps with the Hoechst signal extensively. The scale bar represents a length of 2 µm. b) The indicated zygotes from N2 worms were fixed and stained for H3K9me3 (red) and DNA (blue). Shown below the entire embryos are magnified views of the pronuclei and polar bodies (PB). Note that the H3K9me3 signal overlaps with the Hoechst signal more extensively on the polar bodies than within the pronuclei. The scale bar represents a length of 2 µm. Images are representative of 18 zygotes examined. c) Wild-type 4- and 8-cell embryos were fixed and stained for H3K9me3 (red) and DNA (blue). Shown are the magnified views of P_2_ or P_3_ with its corresponding sister cell. The scale bar represents a length of 2 µm. The graph shows the quantification of H3K9me3 foci per nucleus. d) Animals, either wild-type WMM1 animals (control) or *set-25^n5021^* animals treated with *met-2* RNAi, were allowed to lay embryos. The embryos were then fixed and stained for P-granules (white), RNAPIIpSer2 (red), or DNA (DAPI). The scale bar represents a length of 5 µm.

**Fig. 3.**
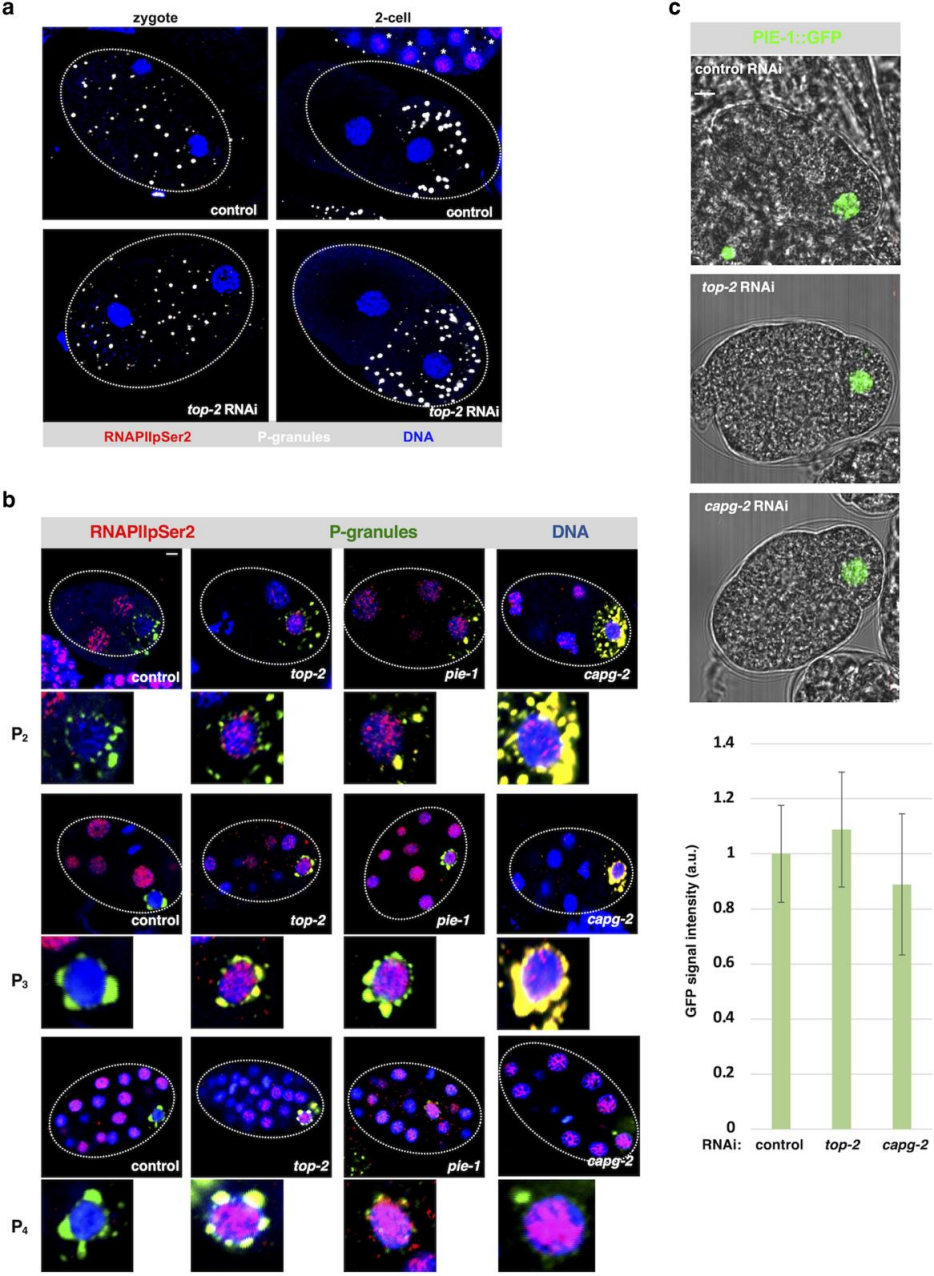
Loss of TOP-2 or CAPG-2 allows for active RNAPII in the P lineage of early embryos. a) 1- and 2-cell embryos that were either exposed to control RNAi (vector only) or *top-2* RNAi were fixed and stained RNAPIIpSer2 (red), DNA (blue), and P granules (white). No RNAPIIpSer2 signals were detected in either P_0_ or P_1_ in any of these samples. We note that a portion of an older embryo is shown at the top of the upper right panel, and these nuclei (marked with an asterisk) clearly contain the RNAPIIpSer2 signals. This shows that the staining procedure worked efficiently. b) Early embryos from N2 worms treated with control, *top-2*, *capg-2,* or *pie-1* RNAi were fixed and stained for RNAPIIpSer2 (red), DNA (blue), and P-granules (green). Shown on top are the entire embryos and the P cell is magnified below. We note that the P granules in this data set would occasionally manifest as a yellow signal, as opposed to the green signal expected, from the combination of primary and secondary antibodies used to detect them. See ‘*Results*’ for an explanation for this. The Scale bar represents a length of 2 µm. c) Fluorescence and phase-contrast microscopy was used to image the living 4-cell embryos expressing PIE-1::GFP after treatment with the indicated RNAi. The graph shows GFP signal quantification across the 3 samples. For signal quantification, confocal slices corresponding to maximal signal intensity were analyzed using ImageJ software to measure pixel density. GFP signal intensity was normalized to control. The number of samples examined for control, *top-2* RNAi, and *capg-2* RNAi was 10, 4, and 6, respectively. Statistical analysis using a Wilcoxon Rank Sum Test revealed no significant differences between the samples. The scale bar represents a length of 5 µm.

RNAPIIpSer2: Rabbit antibody from Abcam (ab5095, Cambridge, Massachusetts) was used at a dilution of 1:100. GFP : mouse Mab #3580, from EMD Millipore, was used at 1:500. FLAG : Mouse antibody F1804 from EMD Millipore was used at 1:1,000. H3K9me2 : Mouse antibody MABI 0307 from Thermo Fisher Scientific was used at a dilution of 1:1,000. H3K9me3 : Rabbit antibody from Abcam (ab176916, Cambridge, Massachusetts) was used at a dilution of 1:1,000. P granules : mouse Mab K76 (Figs. 2d, 3a, and 5a, Supplementary Figs 1 and 3C&D) or mouse Mab OIC1D4 (Fig. 3b), both from the Developmental Studies Hybridoma Bank, were used at a dilution of 1:10 or 1:20, respectively. Secondary antibodies : Alexa Fluor conjugated secondary antibodies from Invitrogen (Thermo Fisher Scientific, Waltham, Massachusetts) were used at a dilution of 1:200.

### Immunofluorescence staining

Adult worms were first washed off plates with 10 ml of M9 minimal medium and washed 3 more times. Then, they were centrifuged at 1.9krpm and the excess medium was removed. Twenty microliters of media containing about 50 worms were spotted on a coverslip and 3 µl of anesthetic (20 mM Sodium Azide and 0.8 M Tetramisole hydrochloride) was added to immobilize them. The worms were dissected using 25Gx5/8 needles (Sigma Aldrich, St. Louis, Missouri). To release early embryos, the worms were cut once midway through their length. To release gonads for oocyte staining, the worms were cut twice, once near the head and once near the tail. The coverslip was then mounted onto poly-L-lysine covered slides and let rest for 5 minutes. The slides were put on dry ice for 30 minutes. The samples were then freeze-cracked by flicking the coverslips off for permeabilization.

For RNAPIIpSer2 staining experiments, once the samples were permeabilized, the slides were put in cold 100% methanol for 2 minutes and then fixing solution (0.08 M HEPES pH 6.9, 1.6 mM MgSO_4_, 0.8 mM EGTA, 3.7% formaldehyde, and 1 × phosphate-buffered saline) for another 30 minutes. After fixing, the slides were washed 3 times with TBS-T (TBS with 0.1% Tween-20) and were blocked for 30 minutes with TNB (containing 100 mM Tris–HCl, 200 mM NaCl, and 1% BSA). The primary antibodies were then applied at the dilutions described above in TNB and the slides were incubated at 4°C overnight.

For H3K9me2, H3K9me3, and GFP staining experiments, the permeabilized samples were put in ice-cold 100% methanol for 10 seconds and then fixing solution (0.08 M HEPES pH 6.9, 1.6 mM MgSO_4_, 0.8 mM EGTA, 3.7% formaldehyde, and 1 × phosphate-buffered saline) for 10 minutes. After fixing, the slides were washed 3 times with TBS-T (TBS with 0.1% Tween-20) and were blocked for 2 hours with TNB (containing 100 mM Tris–HCl, 200 mM NaCl, and 1% BSA) supplemented with 10% goat serum. The primary antibodies were then applied at the dilutions described above in TNB and the slides were incubated at 4°C overnight. On the next day, the slides were washed 3 times with TBS and the slides were incubated with secondary antibodies and Hoechst 33342 dye for 2 hours at room temperature. The slides were washed 3 times with TBS, mounting medium (50% glycerol in PBS), and coverslips were applied and sealed with Cytoseal XYL (Thermo Fisher).

### In situ hybridization chain reaction

A kit containing a DNA probe set, DNA hybridized chain reaction (HCR) amplifier hairpins, and hybridization, wash, and amplification buffers were purchased from Molecular Instruments (molecularinstruments.com). The genes that were examined were *vet-6* and *F58E6.6*. The DNA was visualized with Hoechst 33342 dye. The embryos were prepared by bleaching and were immediately spotted on poly-L-lysine-coated slides. Coverslips were applied and the slides were freeze-cracked to permeabilize the samples. Immediately after freeze-cracking, 500 µl of 100% ice-cold methanol was applied over the samples. The slides were dried off by tilting slides, 1 ml of 4% paraformaldehyde was added and the samples were incubated in a humidity chamber for 10 minutes. The samples were then washed 3 times with 100 µl of PBS-T. A 1:1 solution of probe hybridization buffer (PHB) and PBS-T was added to the samples, and they were incubated for 5 minutes at room temperature. The samples were then prehybridized with PHB for 30 minutes at 37°C and DNA probes (at a final concentration of 2 picomoles per 500 µl of PHB) were added to the samples and were incubated overnight at 37°C. The next day, the samples were washed 4 times with probe wash buffer at 37°C with 15 minutes of incubation for each wash. They were then washed 3 more times with 5xSSCT at room temperature. The samples were preamplified with Amplification buffer for 30 minutes at room temperature. Probe amplifier hairpins were snap cooled by heating to 95°C for 90 seconds and putting in a dark drawer for 30 mins, then they were added to the sample. Worms were incubated with the hairpins overnight in a dark drawer. On the third day, the samples were washed with 5xSSCT and incubated with Hoechst-33342 (1:5,000 dilution) for 15 minutes. Finally, the samples were mounted on poly-L-lysine-coated slides and imaged.

### Immunofluorescent imaging

All slides were imaged using an Olympus Fluoview FV1000 confocal microscope using Fluoview Viewer software. A magnification of 600 × (60 × objective and 10 × eyepiece magnifications) was used. Laser intensity was controlled for experiments to achieve consistency among samples.

## Results

### The H3K9 methyltransferases SET-25 and MET-2 are dispensable for genome silencing in the P lineage

Our previous work on oocytes, spermatocytes, and starved L1 PGCs has revealed a prominent role for the H3K9me heterochromatin pathway in global genome silencing (Belew *et al.* 2021, 2023; Chien and Michael 2023). While the H3K9me heterochromatin pathway has been well studied in *C. elegans* embryos (Towbin *et al.* 2012), to our knowledge there are no published studies focusing on the transcriptional status of the P lineage and how it connects to this pathway. To fill this knowledge gap, we first stained early embryos for the H3K9me3 mark, which is deposited by SET-25 (Towbin *et al.* 2012). One hallmark of silencing in both oocytes and starved PGCs is a large accumulation of H3K9me3 marks on chromatin, as inferred by immunofluorescence microscopy (Belew *et al.* 2021, 2023). In *C. elegans*, the oocytes are named by their position relative to the spermatheca in the tube-shaped gonad, where −1 is closest. We have previously shown that, starting at the −5 position, H3K9me3 marks begin to accumulate on chromatin such that at the −2 and −1 positions H3K9me3 signals overlap with much of the DAPI-based DNA signal (Belew *et al.* 2023). Although these data have been recently published, we have included images of H3K9me3 staining in −2 and −1 oocyte in the current study to allow comparison with our new data on the embryo (Fig. 2a). For a detailed discussion of how this chromatin mark accumulates in oocytes, we refer the reader to our previous work (Belew *et al.* 2023). By contrast to oocytes, in zygotes, the maternal pronucleus (as well as its paternal counterpart) showed greatly reduced H3K9me3 signal intensity, manifesting as small patches on otherwise barren chromatin (Fig. 2b). This was true of both newly fertilized zygotes and those that were nearing mitosis (Fig. 2b). Interestingly, the polar bodies retained strong H3K9me3 signals (Fig. 2b). These data suggest that, upon fertilization, H3K9me3 marks are rapidly erased from maternal chromatin, but this does not occur on polar bodies as they have been ejected from the zygote's cytoplasm and thereby escape erasure. This rapid erasure is reminiscent of what occurs in Z2/Z3, where H3K9me marks disappear within an hour of feeding (Belew *et al.* 2021). We next examined P cells in older embryos, specifically P_2_ and P_3_, and found that the pattern of H3K9me3 marks is very similar to that observed in the somatic cells EMS and C, in that just small patches of signal are present on chromatin (Fig. 2c). We counted the patches, or foci, in these cells and found no difference between P_2_/P_3_ and EMS/C. We conclude that P blastomeres do not accumulate H3K9me3 marks to nearly the same extent as oocytes or starved PGCs.

Our previous work has shown that co-depletion of the SET-25 and MET-2 methyltransferases prevents both H3K9me2 and me3 deposition and genome silencing in starved PGCs (Belew *et al.* 2021). We, therefore, asked if these enzymes are required for transcriptional repression in P blastomeres. For this we used a *set-25* knockout strain (n5021) and treated these animals with RNAi against *met-2*. As shown in Supplementary Fig 1, these conditions resulted in complete loss of detectable H3K9me2 and me3 marks in all P blastomeres, as well as somatic cells. We next monitored these embryos for active RNA polymerase II (RNAPII), and to do so we used immunofluorescence staining with an antibody that detects a phospho-serine 2 epitope on the RNAPII carboxyl-terminal domain (CTD). The presence of this RNAPIIpSer2 signal in the nuclei is a marker for actively elongating RNAPII (Palancade and Bensaude 2003), and we and others have used this approach extensively to identify transcriptionally active nuclei within a variety of *C. elegans* tissues (Seydoux and Dunn 1997; Walker *et al.* 2007; Ghosh and Seydoux 2008; Guven-Ozkan *et al.* 2008; Shakes *et al.* 2009; Bowman *et al.* 2013; Tocchini *et al.* 2014; Butuči *et al.* 2015; Fassnacht *et al.* 2018; Wong *et al.* 2018; Belew *et al.* 2021, 2023; Chien and Michael 2023). The antibody that we are using, a rabbit polyclonal that recognizes RNAPIIpSer2, has previously been validated by us for use in *C. elegans* embryos (Belew *et al.* 2021). The samples were also stained with an antibody recognizing P granules, as these structures are specific to P cells and can therefore be used to distinguish them from somatic cells. For this and all subsequent RNAPIIpSer2 analyses in this paper, nuclei were scrutinized for the appearance of RNAPIIpSer2 signals, and data were tabulated with simple yes or no calls, as others have done in the past (Ghosh and Seydoux 2008). We chose yes/no over quantification of RNAPIIpSer2 immunofluorescence (IF) intensity because we believe that quantifying IF signals can give misleading results owing to sample-to-sample variation in antibody penetration as well as nonlinear signals owing to amplification by secondary antibodies. For control samples, we found that RNAPIIpSer2 signals were absent from both 1- and 2-cell embryos, as expected, and present in somatic nuclei but not P blastomeres in embryos containing P_2_, P_3_, and P_4_ (Fig. 2d and Table 1). For *set-25^n5021^* animals exposed to *met-2*). We conclude that the H3K9me pathway does not play a prominent role in genome silencing in P blastomeres.

**Table 1.** Percent of P blastomeres positive for RNAPIIpSer2.

	P_0_ (*n*)	P_1_ (*n*)	P_2_ (*n*)	P_3_ (*n*)	P_4_ (*n*)
Fig. 2d					
control	0 (7)	0 (9)	0 (10)	0 (10)	0 (11)
*set-25* + *met-2* RNAi	0 (7)	0 (10)	8.3 (12)	0 (10)	18.2 (11)
Fig. 3a					
control	0 (10)	0 (10)	n.a.	n.a.	n.a.
*top-2* RNAi	0 (10)	0 (10)	n.a.	n.a.	n.a.
Fig. 3b					
control	n.a.	n.a.	1.6 (62)	1.1 (88)	6.7 (89)
*top-2* RNAi	n.a.	n.a.	56.1 (41)	50 (52)	57.8 (45)
*pie-1* RNAi	n.a.	n.a.	69.2 (13)	94.4 (18)	93.3 (15)
*capg-2* RNAi	n.a.	n.a.	27.8 (18)	40 (40)	52.4 (21)
Fig. 5a					
control (15°C)	n.a.	n.a.	5 (20)	n.a.	n.a.
control (24°C)	n.a.	n.a.	35 (20)	n.a.	n.a.
*mex-5/6* RNAi (15°C)	n.a.	n.a.	5 (40)	n.a.	n.a.
*mex-5/6* RNAi (24°C)	n.a.	n.a.	2.5 (40)	n.a.	n.a.

### Loss of TOP-2 and condensin II function results in activation of RNAPII in P_2_, P_3_, and P_4_ of early embryos, but not in P_0_ or P_1_

We next turned our attention to the TOP-2/condensin II pathway. For this we again stained embryos for RNAPIIpSer2 and P granules, and we initially examined 1- and 2-cell embryos after exposure to *top-2* RNAi. Previous work has shown that severe depletion of TOP-2 causes extensive issues with nuclear morphology as nuclei become torn and deformed when chromosome segregation fails (Chan *et al.* 2004; Stanvitch and Moore 2008; Bembenek *et al.* 2013). We sought to avoid these effects and thus we used a mild *top-2* RNAi condition (see ‘*Materials and methods*’ for details) where nuclear morphology was superficially normal. To assess the efficacy of the RNAi, we monitored embryonic lethality, as *top-2* is essential for embryogenesis. As stated in the Methods, our *top-2* RNAi conditions routinely resulted in >90% embryonic lethality, showing that the RNAi is indeed effective despite the lack of a torn nuclei phenotype. As shown in Fig. 3a and Table 1, loss of TOP-2 did not prevent transcriptional repression in either 1- or 2-cells embryos. We next examined the remainder of the P lineage, and here we observed that loss of TOP-2 had the opposite effect, it promoted the appearance of active RNAPII in P_2_, P_3_, and P_4_ (Fig. 3b and Table 1). Active RNAPII was also observed in samples depleted of PIE-1, as expected (Fig. 3b and Table 1). Next, we depleted the CAPG-2 subunit of condensin II and found again that RNAPII became activated in the P_2_, P_3_, and P_4_ blastomeres (Fig. 3b and Table 1). Based on these data, we conclude that while TOP-2 is dispensable for transcriptional repression in P_0_ and P_1_, it is required for repression in P_2_, P_3_, and P_4_, and the same is true of condensin II. We note that in Fig. 3b the P granules would occasionally manifest in yellow color, instead of green as would be expected by the combination of primary and secondary antibodies used to detect them. We believe this is due to occasional cross-reactivity between the secondary antibody used to detect RNAPIIpSer2 (Alexa 555 goat antirabbit, Thermo Fisher #A21428) and the primary antibody used to detect P granules (Mab OIC1D4, from ATCC). This was not an issue when we used Mab K76 to detect P granules (see Figs. 2e and 3a) and because P granules are located outside of the nucleus, the occasional cross-reactivity between Alexa 555 goat antirabbit and Mab OIC1D4 did not impact data collection or interpretation.

One plausible explanation for why loss of TOP-2 or CAPG-2 derepresses RNAPII in P blastomeres is that PIE-1 levels are decreased in these cells. It was, therefore, important to check if *top-2* or *capg-2* depletion reduced PIE-1 expression in P_2_–P_4_. To do so we used a strain harboring PIE-1 tagged with GFP at the endogenous locus, referred to as PIE-1::GFP. PIE-1::GFP is expressed normally and retains PIE-1 function (Kim *et al.* 2014; Gauvin *et al.* 2018). We measured GFP signal intensity in living P cells after control, *top-2*, or *capg-2* RNAi, and, as shown in Fig. 3c, PIE-1::GFP expression in P_2_ was not noticeably altered after depletion of TOP-2 or CAPG-2. Thus, the mechanism by which TOP-2/condensin II silences gene expression in the P lineage does not involve the control of PIE-1 expression levels.

### Aberrant expression of EMS-specific genes is observed in the P lineage when TOP-2 and condensin II are depleted

**Fig. 4.**
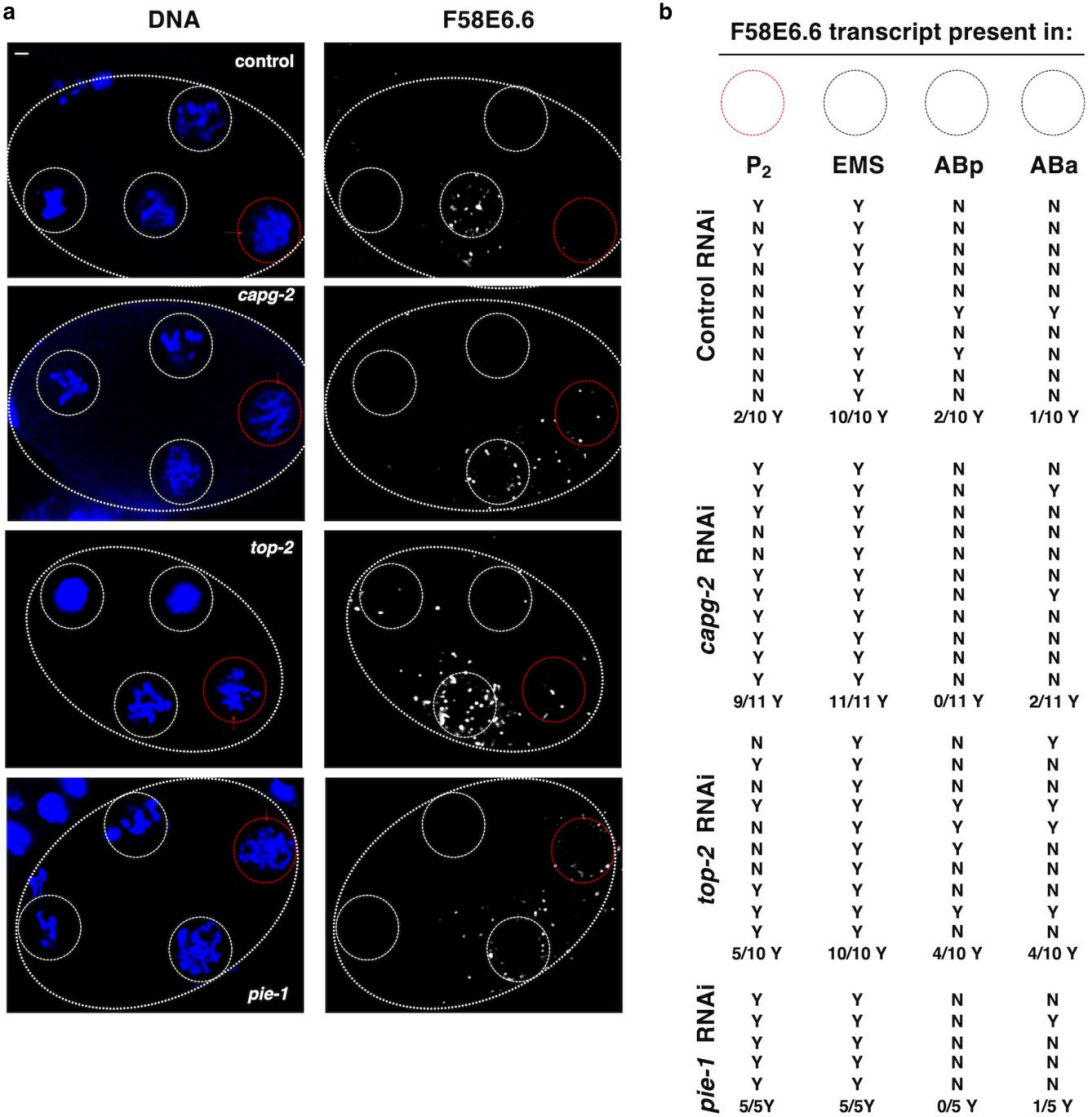
EMS-specific gene is aberrantly expressed in the P_2_ cell of 4-cell embryos after loss of TOP-2 or CAPG-2. a) 4-cell embryos from N2 animals treated with control, *capg-2*, *top-2,* or *pie-1* RNAi were fixed and HCR was performed to probe for *F58E6.6* mRNA (white). DNA was stained using Hoechst-33342 (blue). The representative images are shown. The white dashed circles represent the EMS cell and the red dashed circles represent P_2_. The scale bar represents a length of 2 µm. b) Percentage of samples with *F58E6.6* transcripts in EMS P_2_ (*n* = 40).

Data presented thus far demonstrate aberrant RNAPII activity in the P lineage after depletion of TOP-2 or CAPG-2 (Fig. 3b). To obtain additional evidence of the misregulation of gene expression in these cells, we performed the *in-situ* HCR, also known as RNA-FISH (Choi *et al.* 2016), to detect and localize mRNAs in early embryos. We identified a transcript, *F58E6.6*, that is found predominantly in EMS under normal conditions, with only 10–20% of the wild-type samples showing transcript signals in ABa, ABp, or P_2_ (Fig. 4a, quantification is shown to the right in Fig. 4b, on a per embryo basis). After TOP-2 or CAPG-2 depletion, however, 50% and 82%, respectively, of the embryos expressed *F58E6.6* in P_2_ (Fig. 4a and b). As a positive control, we depleted PIE-1 and found that *F58E6.6* was expressed in P_2_ in 5 out of 5 samples (Fig. 4a and b). These data show that loss of either TOP-2/condensin II or PIE-1 allows unscheduled transcription of *F58E6.6* in P_2_. We also examined another EMS-specific transcript, the previously reported *vet-6* (Seydoux *et al.* 1996). We observed that *vet-6* was also misexpressed in P_2_ upon depletion of TOP-2, CAPG-2, or PIE-1 (Supplementary Fig 2); however, the effect of CAPG-2 depletion was not as pronounced as that seen for the *F58E6.6* transcripts in Fig. 4. Based on these data, we conclude that depletion of TOP-2 and condensin II results in the misexpression of EMS genes in the P lineage of early embryos.

### PIE-1 can repress transcription outside of the P lineage whereas TOP-2 cannot

Our work has shown that genome silencing in the P_2_, P_3_, and P_4_ cells requires both PIE-1 and the TOP-2/condensin II pathway. PIE-1 is expressed exclusively in the P lineage; however, TOP-2 and condensin II are likely expressed in all blastomeres of the early embryo. To confirm this for TOP-2, we used a strain where TOP-2 contains a FLAG tag at the endogenous locus (referred to as TOP-2::FLAG). TOP-2::FLAG retains TOP-2 function (Jaramillo-Lambert *et al.* 2016) and is indeed present in all 4 cells of the 4-cell embryo (Supplementary Fig 3A). This raises the question of why TOP-2 can repress transcription in P cells like P_2_, but not in its somatic sister cell EMS (Fig. 4 and Supplementary Fig. 2). One possibility is that TOP-2 requires the presence of PIE-1 to silence the genome. We have previously shown that this is true in oocytes, where loss of either PIE-1 or TOP-2 prevents transcriptional repression (Belew *et al.* 2023). By contrast, PIE-1 is not expressed in the Z2/Z3 PGCs (Supplementary Fig 3B) and TOP-2 is nonetheless required for genome silencing during L1 starvation (Belew *et al.* 2021). These findings show that TOP-2 can repress transcription through both PIE-1 dependent and independent mechanisms.

To discern the relationship between PIE-1 and TOP-2 in early embryos, we sought a means of misexpressing PIE-1 in the early embryo. Previous work has shown that depletion of the translational regulators MEX-5 and MEX-6 allows PIE-1 expression in all cells of the 4-cell embryo (Schubert *et al.* 2000). The loss of PIE-1 asymmetry in *mex-5/6* RNAi embryos is accompanied by loss of somatic asymmetries as well, e.g. the SKN-1 protein that is normally expressed only in the somatic EMS cell is also found in all four cells after *mex-5/6* RNAi (Schubert *et al.* 2000). Thus, depletion of MEX-5/6 results in blastomeres assuming a merged somatic and germline precursor state. We confirmed the effect of *mex-5/*6 RNAi on PIE-1 localization by using the strain expressing PIE-1::GFP. As shown in Supplementary Fig 3C, in the control samples PIE-1::GFP is expressed solely in P_2_, whereas loss of MEX-5/6 causes a redistribution to all four blastomeres, as expected. Furthermore, when samples were stained for RNAPIIpSer2, control embryos displayed signals in EMS but not P_2_, as expected, while the *mex-5/*6 RNAi samples did not show active RNAPII in any cells, consistent with the misexpression of PIE-1::GFP (Supplementary Fig 3C). Thus, loss of MEX-5/6 causes misexpression of PIE-1::GFP in all blastomeres, and also represses transcription in all blastomeres. To determine if misexpressed PIE-1::GFP is the reason that *mex-5/6* embryos are blocked for transcription, we used a strain expressing both an RNA-binding deficient form of MEX-5 (*mex-5^egx1^*) and PIE-1::GFP (Gauvin *et al.* 2018). This strain is disabled for MEX-5 function but remains viable through MEX-6 activity. We treated this strain with *mex-6* RNAi together with either control or *pie-1* RNAi to create conditions where animals were depleted of either MEX-5/6 activity alone or MEX-5/6 and PIE-1::GFP activity. Transcriptional activity in four-cell embryos was then assessed via RNAPIIpSer2 staining. As shown in Supplementary Fig 3D, in samples treated with *mex-6* plus control RNAi, PIE-1::GFP was expressed in all four blastomeres and RNAPIIpSer2 was attenuated. By contrast, in samples exposed to RNAi against both MEX-5 and GFP-PIE-1, GFP signals were reduced and RNAPIIpSer2signals were easily detected in all four blastomeres. We conclude that the pan-embryo transcriptional repression observed after loss of MEX-5/6 activity is due to misexpression of PIE-1::GFP.

**Fig. 5.**
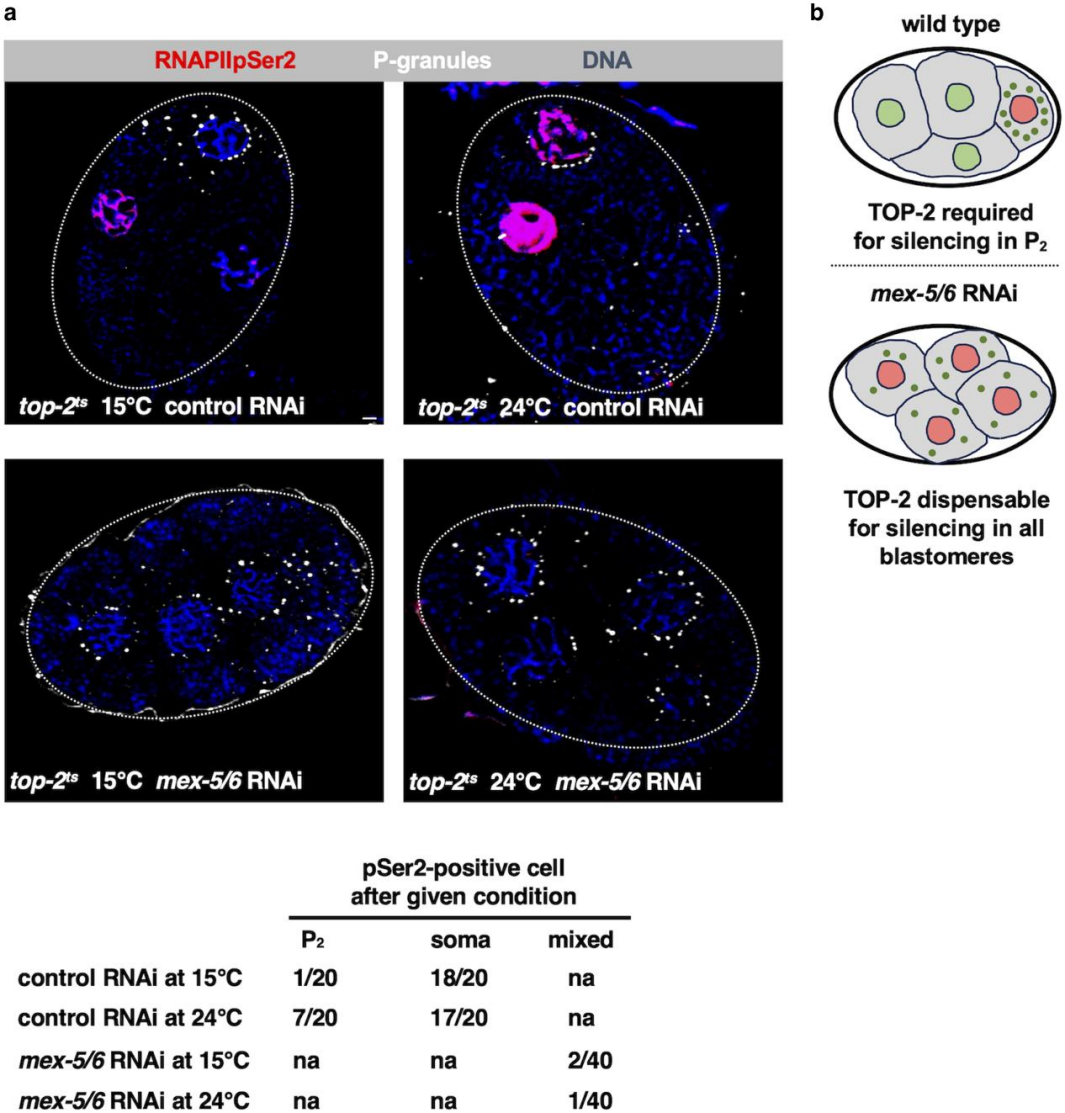
PIE-1 does not require TOP-2 to repress transcription in *C. elegans* early embryos. a) 4-cell embryos from WMM2 (*top-2^ts^*) animals treated with either control or *mex-5/6* RNAi were optionally shifted to the nonpermissive temperature of 24°C for 24 hours or left at the permissive 15°C. Then the samples were fixed and stained for RNAPIIpSer2 (red), DNA (blue), and P granules (white). A total of 20 samples were analyzed over 2 independent replicates. Attenuation of TOP-2 via temperature shift does not reverse the transcriptional silencing by PIE-1 in *mex-5/6* RNAi embryos. Quantification of the data is presented in the following, where “soma” indicates the EMS cell in control RNAi samples. The scale bar represents a length of 2 µm. b) Cartoon of TOP-2 requirement for transcriptional silencing at the 4-cell embryonic stage. Nuclei colored in red indicate transcriptionally silenced cells.

To examine a role for TOP-2 in promoting transcriptional repression by misexpressed PIE-1, we used a temperature-sensitive allele of *top-2* (Jaramillo-Lambert *et al.* 2016) in combination with *mex-5/6* RNAi and examined RNAIIpSer2 in 4-cell embryos at both the permissive temperature (15°C) and the nonpermissive temperature (24°C). For control RNAi, we observed that inactivation of TOP-2 caused the appearance of RNAIIpSer2 in P_2_, as expected, whereas samples raised at the permissive temperature were devoid of RNAIIpSer2 in P_2_ (Fig. 5a upper panels, Table 1). Interestingly, after *mex-5/6* RNAi, transcription was attenuated in all four blastomeres, regardless of the activity state of TOP-2 (Fig. 5a, bottom panels, Table 1). Taken together, our data show that inactivation of PIE-1 in a MEX-5/6-deficient condition prevents pan-embryo transcriptional repression (Supplementary Fig 3D), whereas inactivation of TOP-2 in MEX-5/6-deficient samples does not (Fig. 5a). These findings, in turn, suggest that TOP-2 requires the germline progenitor state for genome silencing in early embryos and that when this state is perturbed, then TOP-2 loses its ability to repress transcription (summarized in Fig. 5b).

## Discussion

In this study, we focused on the early embryonic P lineage with the goal of determining if the TOP-2/condensin II and/or H3K9me/heterochromatin genome silencing pathways are required for transcriptional repression in these cells. Previous work has revealed that two different genome silencers, OMA-1/2 and PIE-1, are active in the P lineage, with OMA-1/2 responsible for P_0_ and P_1_ (as well as AB) and PIE-1 taking over for P_2_–P_4_ (Fig. 1). Our work has added to this profile with the demonstration that TOP-2 and condensin II are needed for efficient silencing in P_2_–P_4_, but not P_0_ or P_1_, and that the H3K9me/heterochromatin pathway is not required at all in the P lineage. We base these conclusions on the following data. For TOP-2/condensin II, we show that depletion of either TOP-2 or the condensin II subunit CAPG-2 results in the appearance of RNAPIIpSer2 signals in P_2_, P_3_, and P_4_ (Fig. 3b) and inappropriate transcription of the somatic *F58E6.6* and *vet-6* genes in P_2_ (Fig. 4 and Supplementary Fig. 2). We note that the impact of TOP-2 or CAPG-2 depletion on transcription in the P lineage was not as profound as depletion of PIE-1 (Fig. 3 and 4, and Supplementary Fig. 2), and this is likely due to the fact that we use mild RNAi conditions for TOP-2/CAPG-2, relative to PIE-1, as stronger conditions for TOP-2/CAPG-2 result in torn, cut, and malformed nuclei, as has been reported previously (Stanvitch and Moore 2008; Bembenek *et al.* 2013). Although we tested just one condensin II subunit in this work, our previous work had shown that depletion of KLE-2, in addition to CAPG-2, disrupts silencing in starved Z2/Z3 (Belew *et al.* 2021), and thus it seems likely that the *capg-2* phenotypes reported here represent a loss of condensin II function and not a condensin II independent function of CAPG-2.

For the H3K9me/heterochromatin pathway, we focused on the SET-25 and MET-2 methyltransferases, which we have previously shown to be required for genome silencing in both oocytes and in starved Z2/Z3 (Belew *et al.* 2021, 2023). We could not reveal a role for these enzymes in P-lineage silencing (Fig. 2). Furthermore, unlike oocytes and starved Z2/Z3, we did not observe a hyper-accumulation of H3K9me3 marks in P-lineage nuclei (Fig. 2), adding further support to the conclusion that the H3K9me/heterochromatin pathway is dispensable for genome silencing in early embryos.

**Fig. 6.**
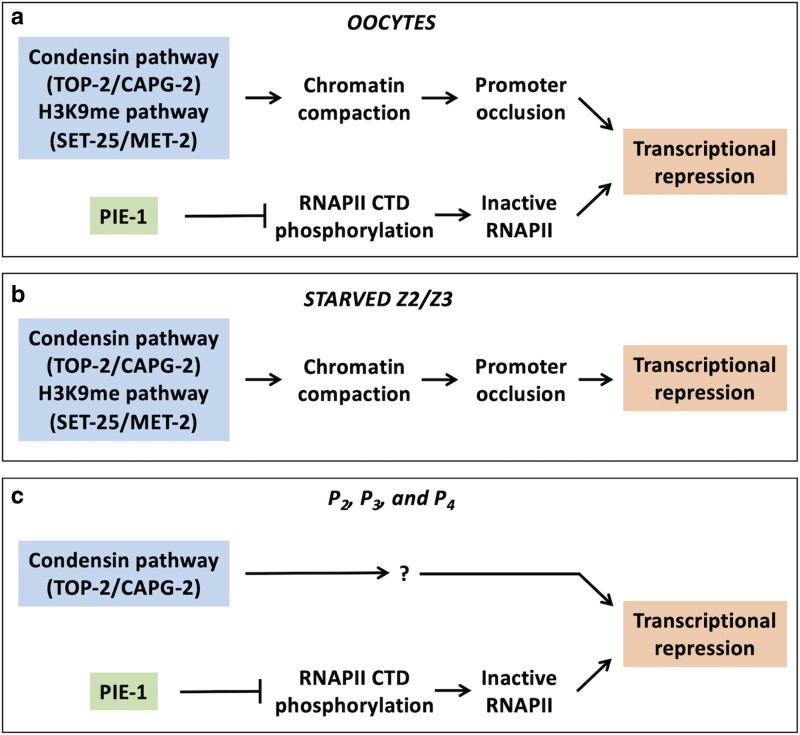
Summary of genome silencing components at different developmental stages in the germline. a) In oocytes, TOP-2/CAPG-2 and the H3K9me pathway repress transcription via chromatin compaction while PIE-1 may work through control of RNAPII CTD phosphorylation. b) In starved L1 larvae, TOP-2/CAPG-2 and the H3K9me pathway are required for transcriptional repression of Z2/Z3. c) In P blastomeres, TOP-2/CAPG-2 works independently of PIE-1 to promote transcriptional repression, although the precise mechanism by which TOP-2/CAPG-2 works is not yet known.

Our work, presented here and elsewhere, has revealed a role for the TOP-2/condensin II pathway in transcriptional repression in three different contexts—oocytes and spermatocytes (Belew *et al.* 2023; Chien and Michael 2023 and Fig. 6a), starved Z2/Z3 PGCs (Belew *et al.* 2021 and Fig. 6b), and in the P_2_, P_3_, and P_4_ cells of early embryos (this work and Fig. 6c). Mechanistically, available data suggest that TOP-2/condensin II silences transcription in oocytes via chromatin compaction, which could block access of the transcriptional machinery to promoter elements (“promoter occlusion” in Fig. 6a). The same mechanism also applies to starved Z2/Z3, as we have shown that starvation triggers extreme chromatin compaction and that when compaction fails, then nuclei remain transcriptionally active (Belew *et al.* 2021 and Fig. 6b). How the TOP-2/condensin II pathway silences transcription in P_2_–P_4_ is not clear as the requirement for DNA replication during cell division seemingly precludes the establishment of a stably compacted chromatin state. Another important question raised by our findings is why, in the early embryo, is TOP-2/condensin II only active for transcriptional repression in P blastomeres, despite being ubiquitously expressed. Indeed, we have shown here that when P-cell identity is lost, then PIE-1 no longer requires the presence of TOP-2 to block transcription, as it does in both the P lineage and in oocytes (this work and Belew *et al.* 2023). Thus, it appears that there is something about the germline or germline progenitor state that transforms TOP-2/condensin II into a genome silencing system. One plausible explanation is that germline and somatic condensin complexes contain different subunits, as has been shown in *Tetrahymena* (Howard-Till and Loidl 2018), or that germline-specific cofactors control condensin activity, as has been shown in *Drosophila* where the vasa protein controls condensin I activity (Pek and Kai 2011). Clearly, further work is needed to understand how TOP-2/condensin II is differentially regulated in germline vs soma in *C. elegans*. In addition, future studies using higher resolution quantitative data may yield new insights.

Lastly, our work has also revealed an intriguing connection between PIE-1 and the TOP-2/condensin II pathway during germline genome silencing. We found that loss of either pathway prevents silencing in both oocytes and in P_2_–P_4_, suggesting that the two systems are dependent on one another as opposed to acting redundantly. The mechanism by which PIE-1 blocks transcription is unclear. Early work suggested that PIE-1 competes with the RNAPII CTD for binding to the cyclin T component of the CDK9-cyclin T complex that is required for phosphorylation and activation of RNAPII (Batchelder *et al.* 1999; Wang and Seydoux 2013). PIE-1 has been proposed to titrate cyclin T away from RNAPII, thereby suppressing the CTD phosphorylations that are needed to trigger RNAPII elongation. More recent work, however, has shown that PIE-1 in the adult germline controls SUMOylation of chromatin remodeling factors, such as the NuRD complex component and the histone deacetylase HDA-1 (Kim *et al.* 2021). Thus, it may be that PIE-1 connects to the TOP-2/condensin II pathway through chromatin as opposed to direct regulation of RNAPII.

## Supplementary Material

jkae236_Supplementary_Data

## Data Availability

Strains and plasmids are available upon request. The authors affirm that all data necessary for confirming the conclusions of the article are present within the paper, figures, and tables. Supplemental material available at G3 online.
