# Supplementary material for: The topoisomerase II/condensin II axis silences transcription during germline specification in Caenorhabditis elegans
Source: G3 (Bethesda). 2024 Oct 3;14(12):jkae236. doi: 10.1093/g3journal/jkae236 (PMC11631511; doi:10.1093/g3journal/jkae236)
Supplement: jkae236_Supplementary_Data [file jkae236_supplementary_data.zip › Figure_S1_G3-2024-405387.docx]

**Figure S1**

**
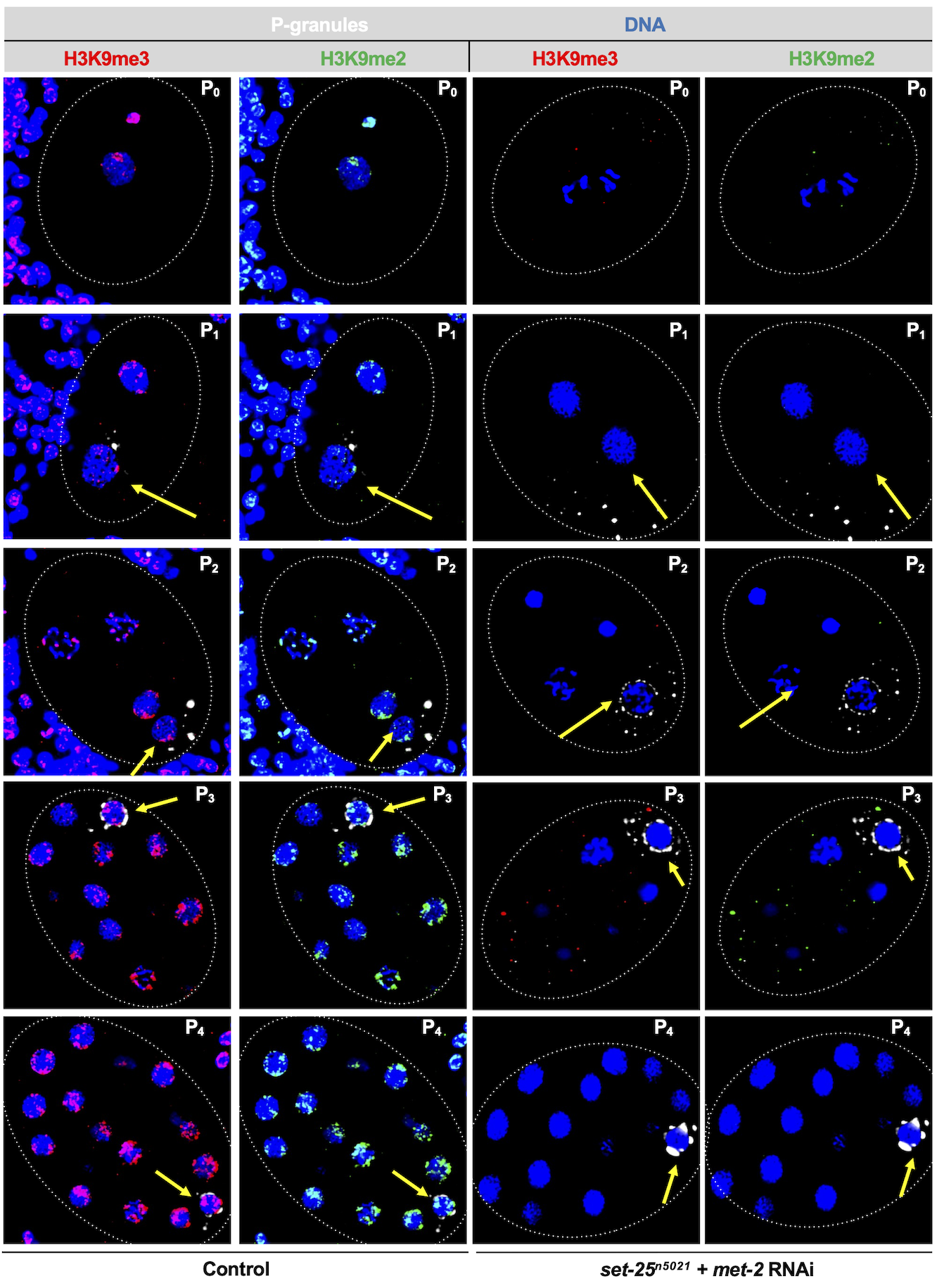
**

**Figure S1: H3K9me2 and -me3 staining in control and methyltransferase-depleted embryos.**

Either wild type (control) embryos or *set-25^n5021^* embryos that had been exposed to *met-2* RNAi were fixed and stained for H3K9me2 (green), H3K9me3 (red), and P granules (white). Shown are representative images from the indicated developmental stages. Note that the H3K9me2 and -me3 signals become undetectable after loss of SET-25 and MET-2, attesting to the efficacy of the depletion. The yellow arrows point to the P blastomere.
