# Supplementary material for: The topoisomerase II/condensin II axis silences transcription during germline specification in Caenorhabditis elegans
Source: G3 (Bethesda). 2024 Oct 3;14(12):jkae236. doi: 10.1093/g3journal/jkae236 (PMC11631511; doi:10.1093/g3journal/jkae236)
Supplement: jkae236_Supplementary_Data [file jkae236_supplementary_data.zip › Figure_S2_G3-2024-405387.docx]

**Figure S2**

**
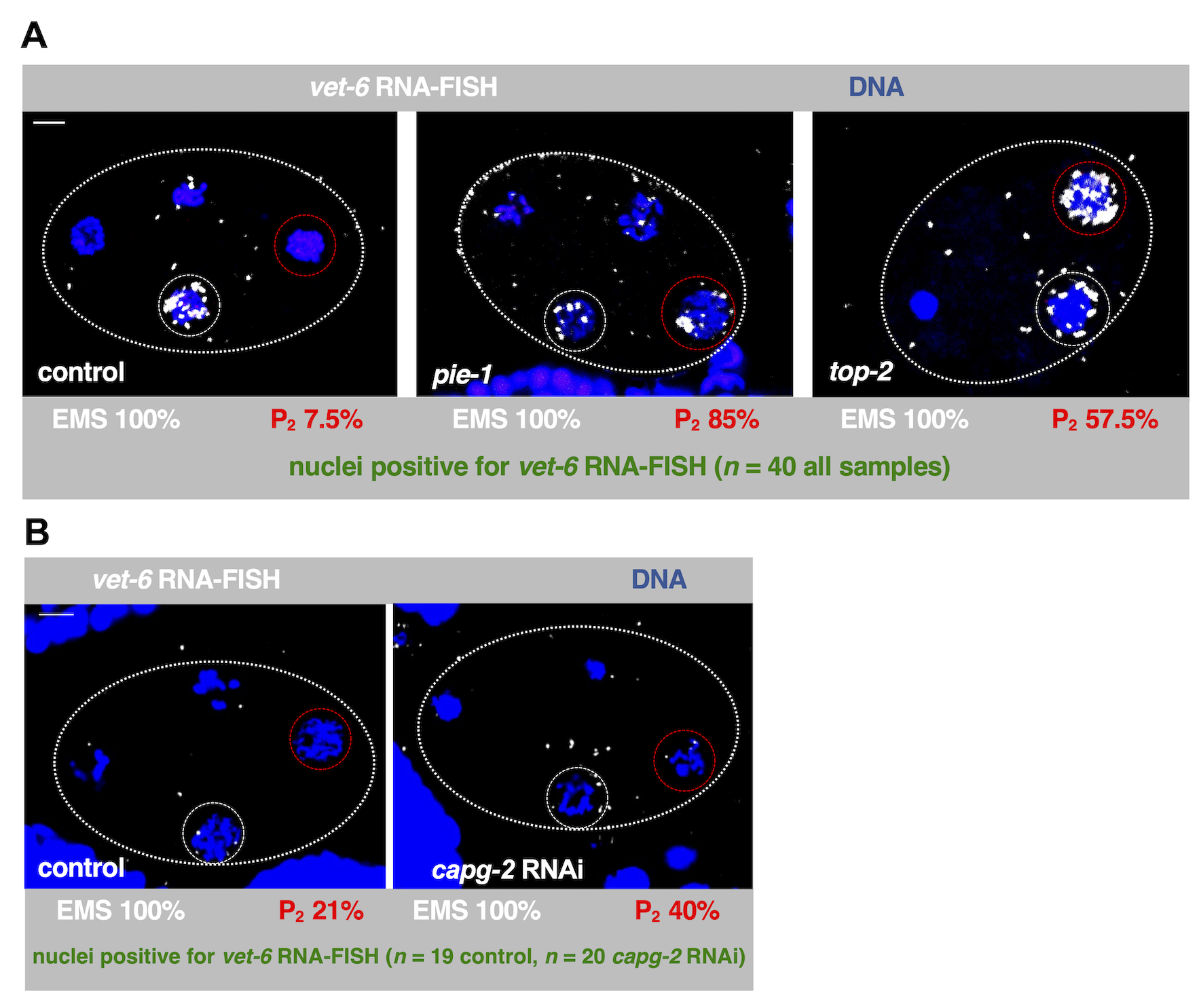
**

**Figure S2: EMS specific gene is aberrantly expressed in the P_2_ cell of 4-cell embryos.**

1. HCR was performed using 4-cell embryos from N2 animals treated with control, *pie-1*, or *top-2* RNAi to probe for *vet-6* mRNA (white). DNA was stained using Hoechst-33342 (blue). P_2_-associated mRNA signal appears after RNAi depletion of *pie-1* and *top-2*. White dashed circles represent the EMS cell and the red dashed circles represent P_2_. Scale bar represents a length of 5 µm.
2. HCR was performed using 4-cell embryos treated with *capg-2* RNAi to probe for *vet-6* mRNA (white). DNA was stained using Hoechst-33342 (blue). White dashed circles represent the EMS cell and the red dashed circles represent P_2_. Scale bar represents a length of 5 µm.
