# Supplementary material for: The topoisomerase II/condensin II axis silences transcription during germline specification in Caenorhabditis elegans
Source: G3 (Bethesda). 2024 Oct 3;14(12):jkae236. doi: 10.1093/g3journal/jkae236 (PMC11631511; doi:10.1093/g3journal/jkae236)
Supplement: jkae236_Supplementary_Data [file jkae236_supplementary_data.zip › Figure_S3_G3-2024-405387.docx]

**Figure S3**

**
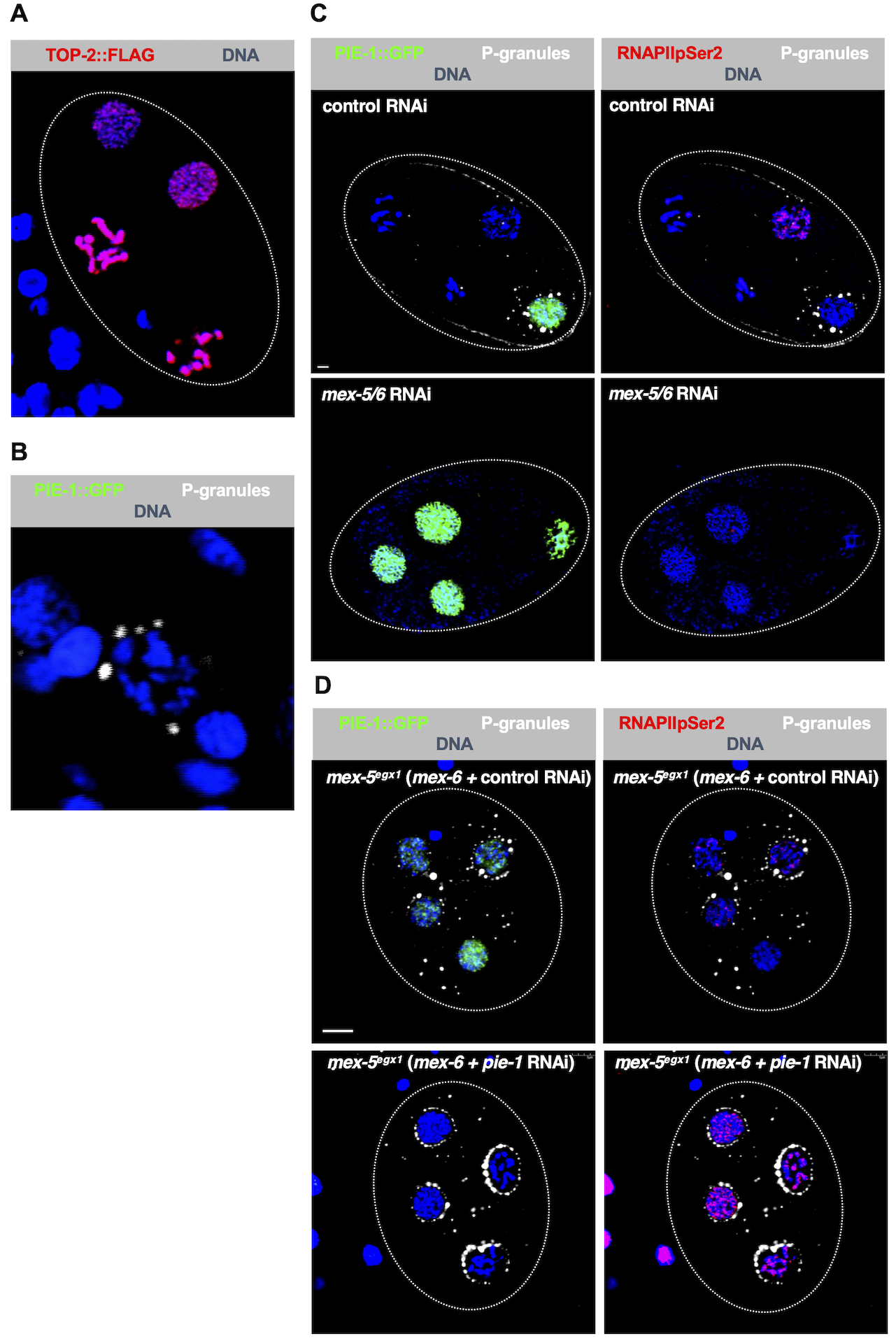
**

**Figure S3: Depletion of MEX-5 and MEX-6 results in PIE-1 expression in all blastomeres of early embryos.**

1. Four-cell AG275 embryo fixed and stained for TOP-2::FLAG (red) and DNA (blue). TOP-2 is distributed across all 4 cells of the embryos. Scale bar represents a length of 2 µm.
2. Z2/Z3 PGC from starved WM330 animals fixed and stained for PIE-1::GFP (green), DNA (blue), and P-granules (white). Scale bar represents a length of 2 µm.
3. Four-cell embryos from WM330 animals optionally treated with either control or *mex-5/6* RNAi were fixed and stained for PIE-1::GFP (green), RNAPIIpSer2 (red), DNA (blue), and P-granules (white). Images of the same embryo stained for PIE-1::GFP and RNAPIIpSer2 are shown side by side. Depletion of *mex-5/6* results in the loss of asymmetric distribution of PIE-1 to P_2_. Scale bar represents a length of 2 µm.
4. Four-cell embryos from EGD175 animals were treated with a combination of either *mex-6*/control or *mex-6/pie-1* RNAi, then fixed and stained for PIE-1::GFP (green), RNAPIIpSer2 (red), DNA (blue), and P-granules (white). *mex-5* mutant embryos depleted of *mex-6* and *pie-1* produce aberrant RNAPIIpSer2 signal in all blastomeres. Scale bar represents a length of 5 µm.
